# Abstracts and Extracts

**Published:** 1887-11

**Authors:** 


### ABSTRACTS AND EXTRACTS.

Professor Theophilus Parvin discusses "Questions in the Treatment of Inevitable Abortion," in *The Medical and Surgical Reporter*, for September 3rd. In cases in which uterine contractions and flow of blood continue for two or three weeks, and there follows arrested development in the uterus and retrograde changes in the mammary glands he thinks abortion is inevitable. In cases of threatened abortion he considers the use of copious hot water vaginal injections a very valuable addition to the employment of rest and opium.

As to methods of emptying the uterus in cases of incomplete abortion he prefers first one or two fingers, and, secondly, Emmet's curette forceps.

At the close of more than a column of discussion of the question of immediately emptying the uterus he writes: "Of course at the time of the miscarriage make it complete if possible without injury to the uterus—let the interference be digital rather than instrumental, unless the former fails and hæmorrhage persists; but that time past and part of the ovum being retained, the os closing, I believe it better to wait until distinct call for action is given. There is a middle ground between immediate intervention and absolute expectancy; and in that ground, my faith is, the path of safety lies."

Acetone and diacetic acid in the urine. See p. 310 *et al.* in *The Medical and Surgical Reporter*, for September 3, 1887.

In *The Medical and Surgical Reporter* of September 3, 1887, Enos T. Blackwell, M. D., discusses the use of tobacco as a relaxant in cases of incarcerated hernia, and reports again two cases first reported in 1860. He used the cut smoking tobacco, binding a mass, about the size of the bowl of a common pipe, about twelve grains in weight, with string, into the form of a suppository. The ends of the string were left long to facilitate the removal of the tobacco when the desired result —reduction of the hernia—had been accomplished. In one of the reported cases the relaxation of the sphincter was so great that the suppository fell out of the rectum.

Dr. Blackwell thinks this method of using tobacco for such effects is thoroughly safe because the suppository can be withdrawn whenever symptoms of intoxication appear, or the desired result is achieved. He considers the use of clysters of the extract very dangerous.

